# Clinical features associated with poor response and early relapse following BCMA-directed therapies in multiple myeloma

**DOI:** 10.1038/s41408-024-01081-z

**Published:** 2024-07-23

**Authors:** Matthew J. Rees, Aytaj Mammadzadeh, Abiola Bolarinwa, Mohammed E. Elhaj, Arwa Bohra, Radhika Bansal, Sikander Ailawadhi, Ricardo Parrondo, Saurabh Chhabra, Amit Khot, Suzanne Hayman, Angela Dispenzieri, Francis Buadi, David Dingli, Rahma Warsame, Prashant Kapoor, Morie A. Gertz, Eli Muchtar, Taxiarchis Kourelis, Wilson Gonsalves, S. Vincent Rajkumar, Yi Lin, Shaji Kumar

**Affiliations:** 1 Division of Hematology, Mayo Clinic, Rochester, MN USA; 2 Division of Hematology, Mayo Clinic, Jacksonville, FL USA; 3 Division of Hematology, Mayo Clinic, Phoenix, AZ USA; 4 Division of Clinical Hematology, Peter MacCallum Cancer Centre & Royal Melbourne Hospital, University of Melbourne, Parkville, VIC Australia

**Keywords:** Myeloma, Risk factors, Cancer immunotherapy, Targeted therapies, Tumour immunology

## Abstract

Three classes of BCMA-directed therapy (BDT) exist: antibody drug-conjugates (ADCs), CAR-T, and T-cell engagers (TCEs), each with distinct strengths and weaknesses. To aid clinicians in selecting between BDTs, we reviewed myeloma patients treated at Mayo Clinic with commercial or investigational BDT between 2018-2023. We identified 339 individuals (1-exposure = 297, 2-exposures = 38, 3-exposures = 4) who received 385 BDTs (ADC = 59, TCE = 134, CAR-T = 192), with median follow-up of 21-months. ADC recipients were older, with more lines of therapy (LOT), and penta-refractory disease. Compared to ADCs, CAR-T (aHR = 0.29, 95%CI = 0.20–0.43) and TCEs (aHR = 0.62, 95%CI = 0.43–0.91) had better progression-free survival (PFS) on analysis adjusted for age, the presence of extramedullary (EMD), penta-refractory disease, multi-hit high-risk cytogenetics, prior BDT, and the number of LOT in the preceding 1-year. Likewise, compared to ADCs, CAR-T (aHR = 0.28, 95%CI = 0.18–0.44) and TCEs (aHR = 0.60, 95%CI = 0.39–0.93) had superior overall survival. Prior BDT exposure negatively impacted all classes but was most striking in CAR-T, ORR 86% vs. 50% and median PFS 13-months vs. 3-months. Of relapses, 54% were extramedullary in nature, and a quarter of these cases had no history of EMD. CAR-T demonstrates superior efficacy and where feasible, should be the initial BDT. However, for patients with prior BDT or rapidly progressive disease, an alternative approach may be preferable.

## Introduction

The B-cell maturation antigen (BCMA) is a transmembrane receptor and member of the tumor necrosis factor receptor superfamily. Essential to plasma cell survival, and minimally expressed on non-hematopoietic tissues, BCMA has emerged as a leading therapeutic target in multiple myeloma (MM) [1–3]. Since 2020, the US Food and Drug Administration (FDA) has approved five BCMA-directed therapies with many other products under investigation, broadly these are divided into three classes; chimeric antigen receptor (CAR)-T cells, T-cell engagers (TCEs) and antibody-drug-conjugates (ADCs). Each BCMA-directed therapeutic class has a role in the treatment of MM, with distinct strengths and weaknesses. The abundance of novel therapeutics means selecting the most appropriate agent for any individual is essential, especially since trial and real-world evidence has consistently shown that the efficacy of BCMA-directed therapies in patients with prior BCMA-exposure is reduced compared to BCMA-naïve individuals [4–7].

While the approved therapies have demonstrated manageable toxicities and impressive overall response rates (ORRs) in relapsed MM, durable responses remain elusive [3, 8, 9]. Data concerning the mechanisms of relapse and the relative efficacy of therapeutic classes amongst high-risk subgroups are scarce. Based on similar therapies in other hematologic conditions, immune system exhaustion and intrinsic tumor resistance via genomic or antigenic changes are the most likely causes of treatment failure [8–10]. Since assays to assess immune-fitness or a tumor’s predilection to antigen loss are not commercially available, we investigated clinical features associated with treatment failure to assist clinicians in selection of the optimal BCMA-class in relapsed myeloma.

## Method

### Study design

The study was approved by the institutional review board of Mayo Clinic. A retrospective study of all MM patients treated at Mayo Clinic with commercial or investigational BCMA-targeted therapies between April 2018 and June 2023. Patients were identified through pharmaceutical dispensing records and the Mayo Clinic’s cellular therapies database. Computerized medical records were reviewed to obtain the following data: patient demographics, baseline disease characteristics, treatment history, disease response, and survival outcomes.

### Definitions

Oligo secretory disease was defined by a serum M-protein <1 g/dL, urine M-protein <200 mg/24 h and serum FLC level <10 mg/dL [11]. Non secretory disease was defined by a normal serum FLC ratio, normal serum and urine immunofixation [12]. Extramedullary disease (EMD) was defined by the presence of visceral or soft tissue myelomatous lesions not contiguous with skeletal lesions. Plasma cell leukemia (PCL) was defined by the presence of ≥5% circulating plasma cells (CTCs) on blood smear examination [13]. Patients were categorized as having double- or multi-hit HRCAs if they had two or more of the following abnormalities identified on FISH either at diagnosis or at the time of progression (if within 3-years of BCMA therapy): del(17p), amplification or gain(1q), del(1p), t(4;14), t(14;20) and t(14;16).

Disease progression and the best treatment response were determined according to the International Myeloma Working Group criteria [14]. Patients were considered refractory to therapy if they achieved no response or progressed on or within 60-days of receiving standard doses of a specific therapy [14]. Triple-class refractoriness was defined as disease refractory to a proteasome inhibitor (PI), immunomodulatory agent (IMID), and an anti-CD38 monoclonal antibody. Penta-class refractoriness was defined as disease refractory to two PI, two IMID, and an anti-CD38 monoclonal antibody.

Prognostic variables including presence of EMD, PCL, beta-2-microglobulin, C-reactive protein, bone marrow plasma cell-burden (BMPC), and peripheral blood absolute lymphocyte count (ALC), collected within 1-month before BCMA treatment commencement. For patients who received CAR-T therapy, the ALC was measured at the time of mononuclear cell apheresis and BMPC was noted within 2-weeks prior to lymphodepletion after bridging chemotherapy.

### Endpoints and statistical analysis

The primary endpoint was progression-free survival (PFS), which was defined from the date of treatment (D_0_ for CAR-T) to the first documentation of disease progression or death. Overall survival (OS) was defined from the date of treatment to the time of death. Forty-two individuals received more than one BCMA-directed therapy at our center, they contributed to analysis for each therapeutic class received. Two patients receiving ADC therapy and one receiving TCE therapy proceeded to an alternate BCMA therapy in the absence of disease progression or intolerance and were censored at the date of next treatment. For the two patients receiving ADC therapy, treatment was used as a bridge to CAR T. For the patient receiving TCE therapy, their clinical trial was halted, and they opted for treatment with a commercial TCE. In 59 cases, patients received a BCMA-directed therapy after previously being treated with an agent from this class, in 13 cases the initial BCMA-directed therapy was delivered at an external center and not captured in this analysis. CAR-T recipients were analyzed on a per protocol analysis, so patients initially planned for CAR-T who did not receive CAR-T infusion were excluded from this study.

Data were analyzed using ‘R’ statistical software, version 4.3.2. Continuous and quantitative discrete variables were described as a median and interquartile range (IQR); categorical variables were described as a number and percentage. A two-sided *P* < 0.05 was considered statistically significant. Group comparisons were performed using the Chi- squared test. Univariate and multivariable analyses of OS and PFS were performed using the Cox proportional hazards regression model. A priori pre-specified prognostic factors included in the adjusted model were EMD or PCL, penta-refractoriness, multi-hit HRCA’s, the number of lines of therapy (LOT) in the preceding 1 year, and prior BCMA-directed therapy. The number of LOTs in the preceding 1 year was selected as a pre-specified covariate because it provides information complimentary to disease refractoriness, namely, the tempo of disease progression, and it was a significant predictor of OS and PFS on univariate survival analysis.

## Results

We identified 339 individuals (1 exposure: *n* = 297, 2-exposures: *n* = 38, 3-exposures: *n* = 4) who received 385 BCMA-directed therapies (ADC = 59, TCE = 134, CAR-T = 192). No patient treated at our institution received the same class of therapy more than once. Patient demographics, disease characteristics and treatment history are summarized, Table 1. One hundred and eighty-eight (49%) treatments were part of a clinical trial, and 121 treatments (31%) included an investigational product or treatment combination which is not FDA approved. Ten individuals received ADC therapy in combination with another agent (IMID = 2, PI = 1, IMID & anti-CD38 = 5, cyclophosphamide = 1, and selinexor = 1). The median duration of follow-up was 21-months (ADCs = 21 months, TCEs = 21 months, CAR-T = 18 months). Patients who received ADC therapy were older, with worse performance status, more prior LOT, and more penta-refractory disease compared to patients who received other forms of BCMA-directed therapy.

**Table 1:** Patient demographics, disease characteristics, and treatment history.

Characteristic	ADC, *N* = 59^a^	CAR T, *N* = 192^a^	TCE, *N* = 134^a^	*p* value^b^
Age (years)	61 (56, 68)	58 (48, 64)	59 (53, 66)	**0.008**
Female	20 (34%)	77 (40%)	55 (41%)	0.6
ECOG				**0.006**
0	13 (34%)	90 (48%)	42 (37%)	
1	18 (47%)	89 (48%)	59 (52%)	
2 or 3	7 (18%)	7 (3.8%)	13 (11%)	
R-ISS 3 at diagnosis	5 (8.5%)	20 (10%)	17 (13%)	0.7
Years from diagnosis	6.6 (3.5, 9.9)	6.0 (3.5, 9.6)	6.1 (4.2, 9.0)	0.8
Double hit HRCA	6 (10%)	43 (22%)	25 (19%)	0.11
EMD/PCL prior to BCMA	17 (36%)	54 (29%)	46 (38%)	0.3
Triple class refractory	50 (85%)	149 (78%)	113 (84%)	0.2
Penta class refractory	34 (58%)	61 (32%)	43 (32%)	**<0.001**
Number of LOTs				**<0.001**
≤4	10 (17%)	80 (42%)	38 (28%)	
5–6	15 (25%)	58 (30%)	49 (37%)	
7–8	19 (32%)	32 (17%)	27 (20%)	
≥9	15 (25%)	22 (11%)	20 (15%)	
Number of LOTs in preceding 1-year				0.063
≤1	22 (37%)	78 (41%)	67 (50%)	
2–3	27 (46%)	100 (52%)	54 (40%)	
≥4	10 (17%)	14 (7.3%)	13 (9.7%)	
BCMA exposed	13 (22%)	16 (8.3%)	30 (22%)	**<0.001**

Bold values: statistically significant *p* value.

^a^Median (IQR); *n* (%).

^b^Kruskal-Wallis rank sum test; Pearson’s Chi-squared test; Fisher’s exact test.

**Fig. 1:**
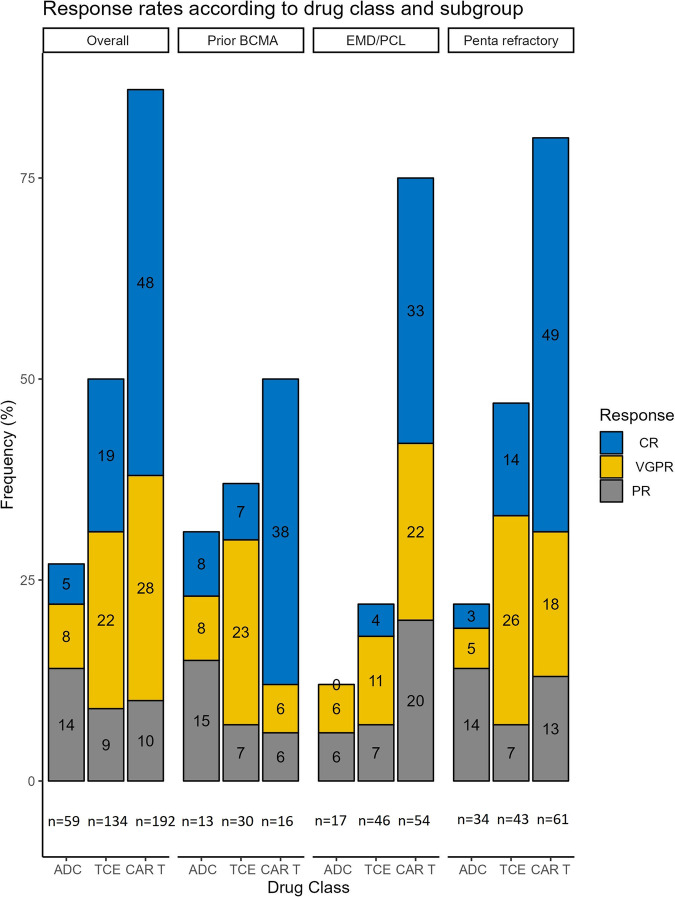
Response rates overall and according to therapeutic class and high-risk subgroups. ADC antibody drug conjugate, TCE T cell engager, CAR-T chimeric antigen receptor T cell, CR complete response, VGPR very good partial response, PR partial response, EMD/PCL extramedullary disease or plasma cell leukemia.

**Fig. 2:**
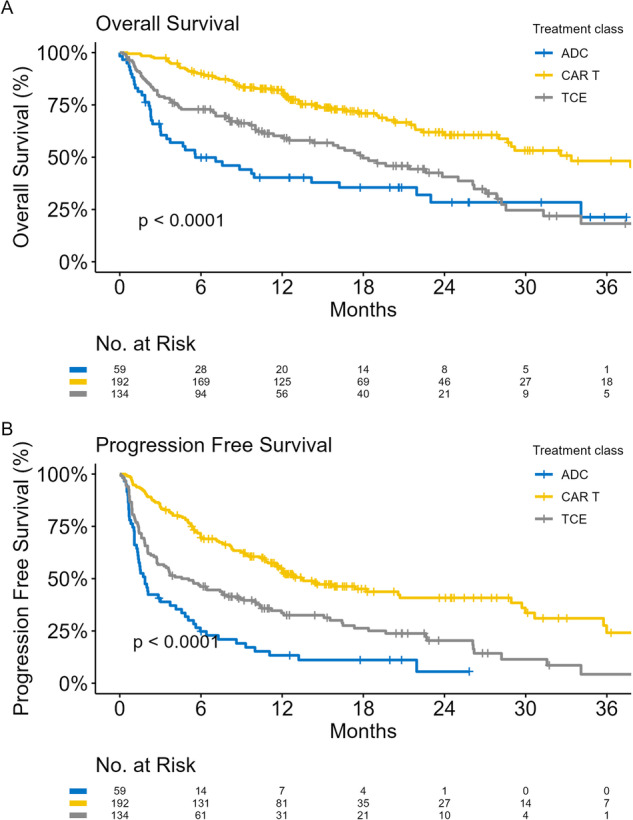
Survival according to the class of BCMA directed therapy. **A** Overall survival according to the class of BCMA-directed therapy. **B** Progression-free survival according to the class of BCMA-directed therapy. Stratified according to the class of BCMA-directed therapy received: ADC (blue), CAR-T (yellow) and TCE (grey). ADC antibody drug conjugate, TCE T cell engager, CAR-T chimeric antigen receptor T cell.

ORRs and response rates according to high-risk subgroups are shown in Fig. 1. PFS and OS according to therapeutic class are shown in Fig. 2. The median PFS was 1.9, 4.6, and 13.4 months for ADCs, TCE, and CAR-T respectively. The median OS was 5.6, 18, and 33.4 months respectively. CAR-T and TCE therapy were associated with superior survival compared to ADCs on multivariable models of PFS and OS adjusted for age, EMD/PCL, penta-refractory disease, double-hit HRCA, prior BCMA-therapy, and the number of LOT in the preceding 1 year, Table 2.

**Table 2:** Multivariable cox proportional hazards model of variables associated with overall and progression free survival.

Characteristic	Overall Survival			Progression-free Survival		
	HR^a^	95% CI^a^	*p* value	HR^a^	95% CI^a^	*p* value
BCMA drug class			**<0.001**			**<0.001**
ADC	—	—		—	—	
CAR T	0.28	0.18, 0.44		0.29	0.20, 0.43	
TCE	0.6	0.39, 0.93		0.62	0.43, 0.91	
Age (years)	1.02	1.01, 1.04	**0.004**	1.01	1.0, 1.02	0.2
EMD or PCL	3.13	2.23, 4.39	**<0.001**	2.49	1.87, 3.31	**<0.001**
Penta-refractory disease	1.23	0.87, 1.73	0.2	1.49	1.12, 1.99	**0.007**
Double-hit HRCA	1.76	1.23, 2.54	**0.003**	1.48	1.08, 2.02	**0.017**
Prior BCMA therapy	0.82	0.52, 1.29	0.4	0.75	0.51, 1.10	0.14
LOT in preceeding 1 year			0.14			0.2
≤1	—	—		—	—	
2–3	1.19	0.84, 1.67		1.26	0.95, 1.68	
≥4	1.67	1.01, 2.74		1.28	0.82, 1.97	

Bold values: statistically significant *p* value.

^a^*HR* hazard ratio, *CI* confidence interval.

The most common commercial products were ciltacabtagene autoleucel (cilta-cel, *n* = 58), idecabtagene autoleucel (ide-cel, *n* = 116), and teclistamab (*n* = 41), baseline disease and treatment characteristics are shown, Supplementary Table 1. Cilta-cel led to superior PFS compared to ide-cel with median PFS of 30 vs 11-months (HR = 0.42, 95%CI 0.25–0.69, *p* < 0.001), Supplementary Fig. 1. Cilta-cel also resulted in superior OS compared to ide-cel with median OS of not-reached vs 29-months (HR = 0.47, 95%CI 0.24–0.93, *p* = 0.031). Of patients treated with teclistamab 61% (25/41) had previously received BCMA-directed therapy. At a median follow up of 8.8 months the median OS and PFS for teclistamab were not-reached and 3.3-months, respectively. When only patients with no prior BCMA therapy who received teclistamab were considered, then PFS at 12-months was 56%. When only patients who responded to teclistamab were considered (CR = 6, VGPR = 9, PR = 3), then PFS at 12-months was 81%.

**Fig. 3:**
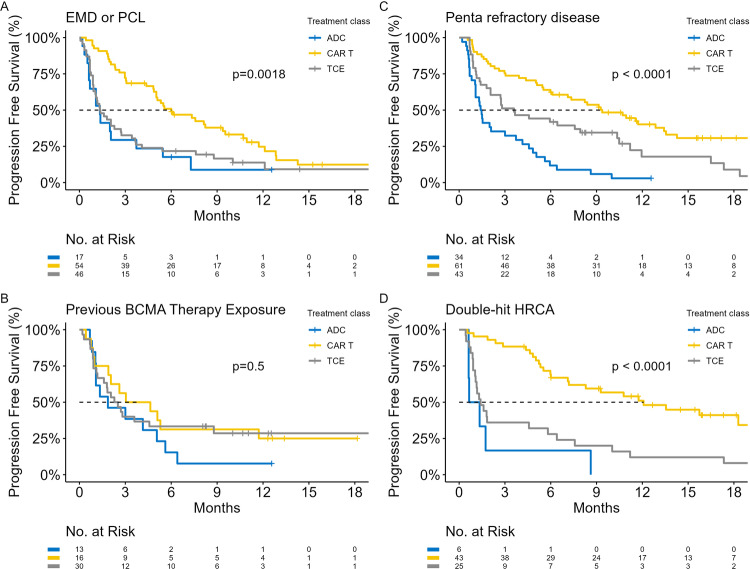
Progression-free survival according to therapeutic class in high-risk subgroups. **A** EMD or PCL, **B** previous BCMA-therapy exposure, **C** penta-refractory disease and **D** double-hit HRCA. Stratified according to the class of BCMA-directed therapy received: ADC (blue), CAR-T (yellow) and TCE (grey). ADC antibody drug conjugate, TCE T cell engager, CAR-T chimeric antigen receptor T cell, EMD extramedullary disease, PCL plasma cell leukemia, HRCA high-risk cytogenetic abnormality.

PFS stratified according to therapeutic class and high-risk subgroups are shown, Fig. 3. In 59 (15%) episodes of BCMA-treatment there was a history of prior BCMA-directed therapy; CAR T only = 32, ADC only = 14, TCE only = 5, TCE & ADC = 2, CAR-T & ADC = 6. In patients who received ≥2 BCMA-treatments, the median time from initial to subsequent BCMA-therapy was 13.6-months (IQR: 6.1, 21.4), and this was not associated with OS (*p* = 0.80) or PFS (*p* = 0.48), irrespective of the previous class of BCMA-therapy. Besides recognized high-risk subgroups, we examined the impact of several clinical covariates associated with immune fitness and disease burden on PFS, Table 3. Increased time (≥24-months) since previous alkylator therapy and had significant positive association on the PFS of CAR-T and TCE, but not ADC recipients. Patients who received alkylator therapy within 24-months compared to ≥24-months of BCMA-directed therapy had comparable performance status (*p* = 0.3), double-hit HRCA (22% vs. 29%, *p* = 0.3), penta-refractory disease (43% vs. 33%, *p* = 0.3), number of LOTs (*p* = 0.11) and prior BCMA exposure (23% vs. 16%, *p* = 0.3). However, patients with alkylator therapy within 24-months had a significantly higher rate of EMD or PCL at the time of BCMA therapy (50% vs. 25%, *p* = 0.006), which may account for the poorer PFS of this group.

**Table 3:** Univariate cox proportional hazards model of variables associated with progression free survival.

Characteristic	All patients				CAR-T				TCE				ADC			
	*N*	HR^a^	95% CI^a^	*p* value	*N*	HR^a^	95% CI^a^	*p* value	*N*	HR^a^	95% CI^a^	*p* value	*N*	HR^a^	95% CI^a^	*p* value
≥24-months since alkylator therapy	161	0.24	0.15, 0.40	**<0.001**	85	0.33	0.17, 0.63	**<0.001**	47	0.1	0.02, 0.42	**0.002**	29	0.4	0.12, 1.37	0.14
≥60-months since ASCT	321	0.77	0.58, 1.01	0.055	164	0.88	0.58, 1.33	0.5	113	0.51	0.33, 0.80	**0.003**	44	0.86	0.46, 1.61	0.6
Abs lymphocyte count ≥0.5 × 10^9/L	365	0.75	0.57, 0.99	**0.043**	188	0.73	0.47, 1.11	0.14	129	0.99	0.61, 1.61	>0.9	48	0.59	0.31, 1.12	0.1
LDH elevated	320	1.82	1.39, 2.39	**<0.001**	182	1.81	1.22, 2.68	**0.003**	104	2.31	1.48, 3.60	**<0.001**	34	1.6	0.75, 3.39	0.2
High Dx burden [BMPC ≥ 50%, CRP ≥ 50 OR B2M ≥ 5]	385	1.5	1.17, 1.94	**0.002**	192	1.99	1.34, 2.96	**<0.001**	134	1.33	0.89, 1.97	0.2	59	1.38	0.71, 2.70	0.3

Bold values: statistically significant *p* value.

^a^*HR* hazard ratio, *CI* confidence interval

The median duration of ADC and TCE therapy was 1.9 months (IQR: 1.1, 4.8) and 3.6 months (IQR: 1.3, 10.3), respectively. The most common reasons for ADC treatment discontinuation were disease progression (64%), followed by intolerance (24%), and death on therapy (5%). The most common reasons for TCE treatment discontinuation were disease progression (79%), followed by intolerance (11%), and completion of therapy or trial closure (6%).

In 86% of relapses (201/234), the presence or absence of EMD was known either through cross-sectional imaging or clinical examination. Fifty-four percent (108/201) of relapses were extramedullary (extraosseous or PCL) in nature, in 27 cases of extramedullary relapse (25%) there was no prior history of EMD/PCL at prior relapse or at diagnosis. The rates of acquired extramedullary relapse were comparable (*p* = 0.69) between therapeutic classes: ADCs 11% (4/36), CAR-T 16% (14/89), and TCEs 12% (9/76). In 97% of relapses (227/234), urine and serum M-spike results were available. In 12% (27/227) of relapses patients converted from a secretory to an oligo- or non-secretory phenotype, and this differed significantly (*p* = 0.020) between therapeutic classes: ADC 2% (1/44), CAR-T 18% (17/93) and TCE 10% (9/90).

**Fig. 4:**
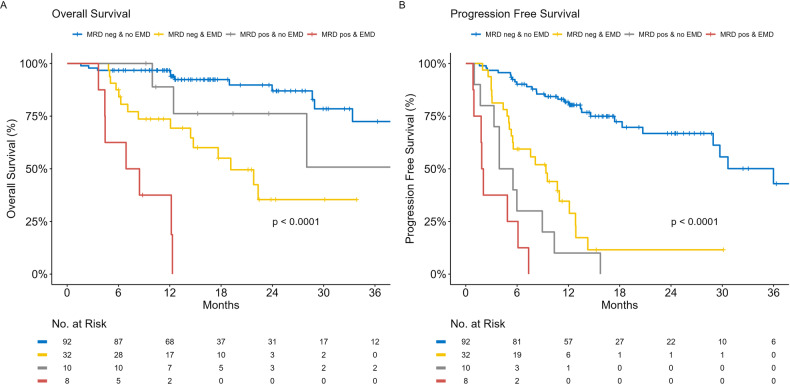
Survival according to measurable residual disease and extramedullary disease status. **A** Overall survival according to measurable residual disease and extramedullary disease status. **B** Progression-free survival according to measurable residual disease and extramedullary disease status. Stratified according to patients who were MRD negative with no EMD (blue), MRD negative with EMD present at the time of BCMA therapy initiation (yellow), MRD positive with no EMD (grey) and MRD positive with EMD present at the time of BCMA therapy initiation (red). MRD measurable residual disease, EMD extramedullary disease, pos positive, neg negative.

Bone marrow MRD assessment, with a sensitivity of 1 × 10^-5^ using flow cytometry was routinely undertaken in CAR-T recipients. Given the high proportion of EMD and the propensity to extramedullary relapse, we compared OS and PFS for patients who had at least one MRD assessment post therapy according to the presence of EMD prior to therapy, Fig. 4. Patients with EMD or PCL who obtained BM MRD negativity status had PFS and OS comparable to patients without EMD or PCL who had BM MRD positivity. Too few patients who received TCE or ADC therapy underwent MRD assessment to permit this same analysis.

## Discussion

In this review we identify several key themes of BCMA-directed therapy. While contingent on myriad other factors, BCMA-directed CAR-T produces superior response rates, PFS, and OS compared to ADC and TCE therapy. EMD is recalcitrant to all BCMA treatment modalities, less reliably prognosticated with BM MRD assessment, and extramedullary relapse post BCMA-directed therapy is common. Lastly, previous BCMA-directed therapy adversely impacts all classes but has the greatest detrimental impact on CAR-T outcomes.

Each BCMA-directed drug class has a role, as reflected in their divergent application to clinical practice. CAR-T recipients are generally younger, anti-BCMA therapy naïve, with good performance status, fewer LOT, and less penta-refractory disease, while the reverse is true for patients treated with ADCs. This suggests CAR-T has been the preferred initial BCMA-directed therapy in relapsed patients, and these more favorable patient and disease characteristics undoubtedly contribute to the observe differences in outcome. When BCMA-directed therapies are compared, CAR-T produces superior survival overall and amongst the high-risk subgroups of EMD/PCL, penta-refractory disease, and double-hit HRCA. While consistent with other reports [7, 15–18], clinicians should interpret this in the appropriate clinical context.

Median PFS following prior BCMA-directed therapy is poor for all classes, however, the negative impact of prior exposure is most striking amongst CAR-T recipients where the ORR declines from 86% to 50% and median PFS declines from 13-months to 3-months. Clinical trial and real-world data indicate the first BCMA-directed treatment is the most effective, and that CAR-T outcomes are most significantly compromised with delayed use [4–6, 19]. In CARTITUDE-2C, patients with prior noncellular BCMA-therapy who received cilta-cel had a substantially lower ORR of 60% and median PFS of 9 months compared to BCMA-naïve patients in CARTITUDE-1 who had an ORR of 98% and median PFS of 35-months [4, 20]. Likewise, the US Myeloma CAR-T cell consortium found prior BCMA-exposure reduced the ORR (74% vs 88%) and median PFS (3 vs 9 months) for ide-cel [5]. Conversely, in MajesTEC-1C, teclistamab obtained an ORR of 45% in patients with prior BCMA-directed CAR-T, comparable to the ORR of 62% in BCMA-naïve patients treated in MajesTEC-1 [6, 15].

A study from Mount Sinai of relapsed patient following BCMA-directed CAR-T, demonstrated superior outcomes with TCEs (including BCMA-directed agents) compared to salvage with conventional doublet, triplet or quadruplet combinations of IMID, PI and anti-CD38 antibodies, with an ORR of 75% vs. 32%, and median PFS of 9.1 vs. under 4.5-months [21]. Similar results demonstrating superior outcomes with BsAb therapy compared to cytotoxic and conventional MM therapy following Ide-cel therapy have been reported in a collaboration from 11 US centers [22]. Antigen escape and an altered T-cell repertoire following sustained TCE exposure may explain the deterioration in efficacy observed with later sequencing of CAR-T [9, 23]. Treatment free periods may mitigate the latter effect [24], and a longer interval between TCE therapies has been associated with improved ORRs to talquetamab [25].

Of the commercially available CAR-T products, cilta-cel produced superior OS and PFS compared to ide-cel. While significant differences existed in the patients treated, including more triple-class and penta-class refractory disease in the ide-cel arm, these results are consistent with; KarMMa-3 where ide-cel had median PFS of 12-months in patients with 2–4 previous LOTs, and CARTITUDE-4 where cilta-cel had 12-month PFS of 76% in lenalidomide refractory patients [16, 17]. A key difference in the cilta-cel construct is the inclusion of two BCMA binding domains, which may contribute to the variation observed in the depth and duration of responses. In contrast to the MajesTEC-1 trial, where teclistamab obtained an ORR of 62% and median PFS of 11 months, we observed more a more sobering ORR of 44% and median PFS of 3.3 months, consistent with other real-world data [15, 26]. Notably, the majority of teclistamab patients included in our study would not have been eligible for MajesTEC-1 due to prior BCMA-exposure, and the survival of BCMA-naïve patients in our study was comparable to MajesTEC-1.

T cell fitness is a critical determinant of CAR-T and TCE efficacy with a less pronounced influence on ADCs, since the latter possesses both immune-independent mechanisms of cytotoxicity [23, 27]. Increased time between alkylator therapy and receipt of BCMA-directed therapy is associated with improved PFS for CAR-T and TCEs, and possibly reflects improved T-cell recovery following cytotoxic therapy. Correlative analysis from the KarMMa trials has previously reported improved PFS with alkylator therapy >6-months before CAR-T administration [28]. Low T-cell numbers including limited CD8+ naïve T-cells, as well as an abundance of regulatory T-cells, terminally exhausted CD8+ T-cells, and T-cells expressing CD38+, PD-1, and TIM-3 are associated poorer responses to MM immunotherapy [23, 29]. Consistent with this, a high pre-treatment ALC, a coarse surrogate for T-cell quantity, was associated with improved PFS in our cohort.

EMD is a high-risk MM subgroup with median OS under a year, increased prevalence in later disease stages, and few effective therapeutic options [30, 31]. Belantamab mafodotin had a disappointing ORR of 6–9% for EMD in DREAMM-2, similarly, ide-cel and cilta-cel had significantly worse median PFS for EMD, at 4.9 and 8.1 months respectively [18, 32, 33]. A meta-analysis of bispecific antibodies in EMD has also shown a reduced ORR of 48%, and survival data is awaited [34]. High levels of soluble BCMA (sBCMA) and the “*sink effect*” may explain the negative impact of disease burden and EMD on BCMA-directed therapy [15, 18, 19, 35]. In vitro sBCMA attenuates the binding and activity of BCMA therapies [36, 37], likewise sBCMA levels correlate with poor response rates [29, 38]. Combination therapy with gamma-secretase inhibitors, which lower sBCMA levels, increase tumor lethality from CAR-T and TCEs in pre-clinical models [39, 40], and have produced encouraging response rates clinically [41–43]. Dual antigenic targeting, such as in the RedirecTT-1 with combination teclistamab and talquetamab, appears to be another effective strategy obtaining an ORR of 83% in EMD. The reduced efficacy of non-BCMA therapies, such as talquetamab in EMD and high-burden disease, suggests sBCMA independent factors such as a lower effector to target ratio or impaired T cell infiltration of large lesions are also relevant [44, 45].

EMD reduces the prognostic significance of BM MRD negativity. While recent data has suggested EMD is not associated with inferior survival in MRD negative patients [46], we observed BM MRD negative patients with EMD experienced inferior survival compared to BM MRD positive patients without EMD. The IFM/DFCI 2009 and Total Therapy 4–6 studies have previously reported the discordance between BM MRD and focal myeloma lesions in newly diagnosed MM. In the former, 14 of 86 patients were PET-CT positive at the same time they were BM MRD negative, and similarly, in the Total Therapy studies, 5 of 42 patients had focal lesion on PET/CT or whole-body diffusion-weighted MRD despite BM MRD negativity [47, 48]. In conjunction with the high rate of extramedullary relapse observed post-immunotherapy, this underscores the importance of combined assessments following BCMA-directed therapy.

This study is subject to all the limitations inherent to a single institution multi-center retrospective review. Significant bias exists in the selection of therapeutics, and undoubtedly contributes to the survival differences observed in this highly heterogenous population. Many important treatment considerations were not included, namely cost, toxicity, and drug-access. Approximately a third of patients received investigational products which do not have proven efficacy. Treatments were categorized according to their underlying mechanism, agnostic of the significant intra-class variation that exists. CAR-T survival was calculated from the date of infusion, as opposed to the date of apheresis as is standard for clinical trials. Lastly, follow up was limited and data on some variables was missing introducing potential non-random error.

While CAR-T demonstrate superior efficacy, when evaluating BCMA-directed therapies physicians need to consider an individual’s preference, medical comorbidities, disease tempo, and treatment history. For patients with rapidly progressive disease and prior BCMA exposure, alternative therapies may be as effective and more practical to deliver than CAR-T. Where feasible, positioning CAR-T as the initial BCMA-directed therapy, and reserving TCEs (BCMA-directed and other) following CAR-T relapse appears to be the most promising sequencing strategy. Extramedullary relapse is common and combining conventional MRD assessments with advanced imaging may lead to earlier detection of relapse. Lastly, the high prevalence and recalcitrant nature of secondary EMD in relapsed populations, stresses the need for combination therapy incorporating BCMA-directed agents at earlier lines of treatment, prior to resistant disease evolution.

### Supplementary information

Supplementary material

## Data Availability

The data generated in this study is available upon reasonable request from the corresponding author.
