# Supplementary material for: Clinical features associated with poor response and early relapse following BCMA-directed therapies in multiple myeloma
Source: Blood Cancer J. 2024 Jul 23;14(1):122. doi: 10.1038/s41408-024-01081-z (PMC11266661; doi:10.1038/s41408-024-01081-z)

Supplementary material

**Table 1.** Baseline patient, disease, and treatment characteristics for recipients of standard of care therapies.


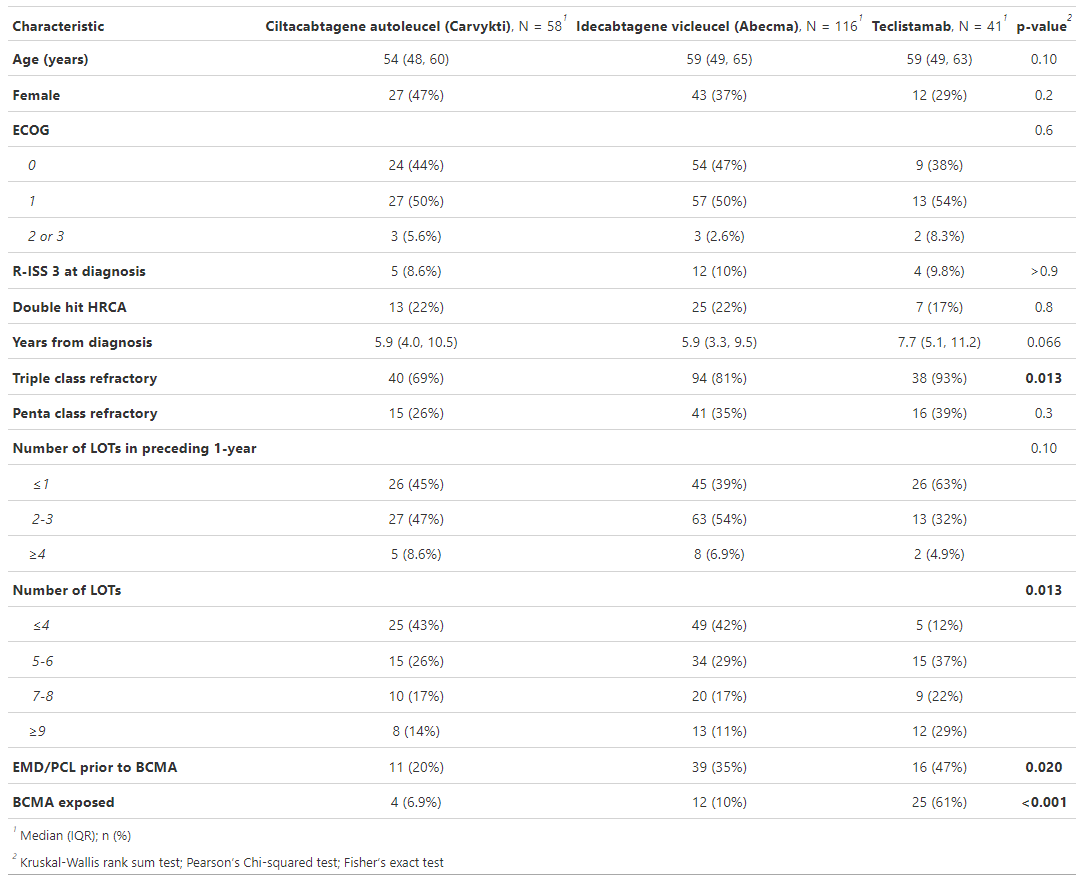


**Supplementary Figure 1.** OS and PFS for cilta-cel and ide-cel.


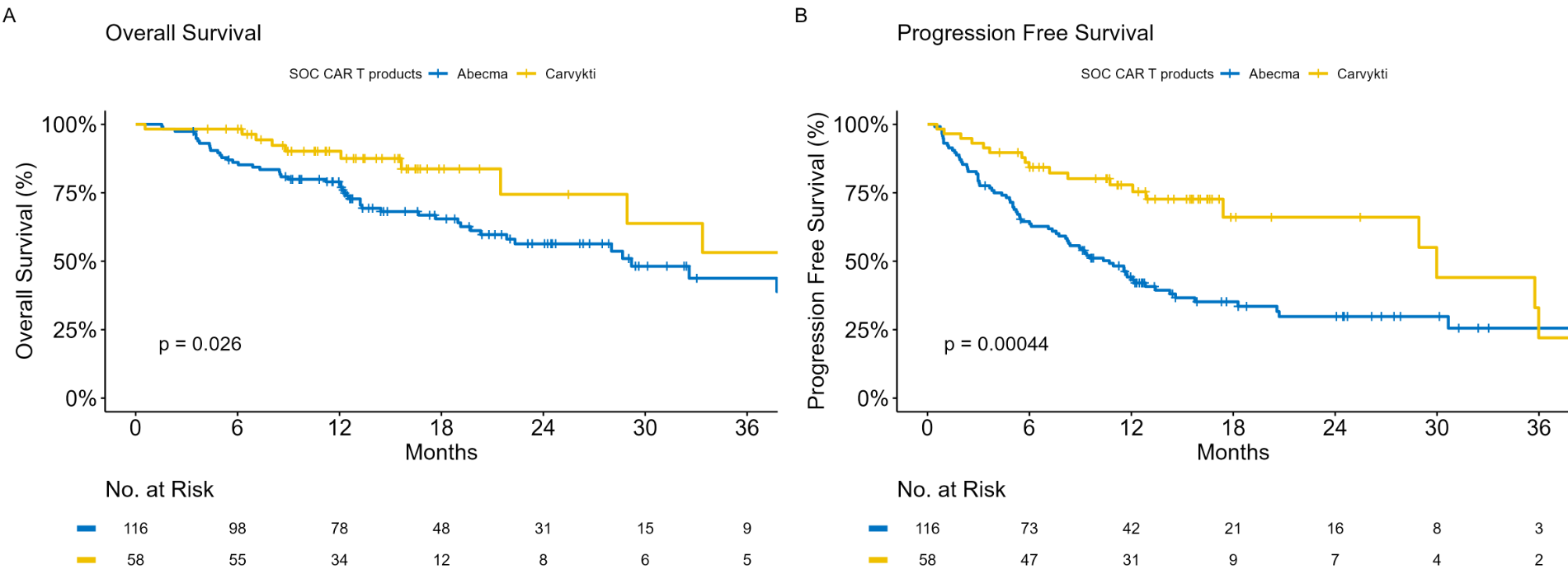

Supplement: Supplementary file 1 — Supplementary material [file 41408_2024_1081_MOESM1_ESM.docx]
